# FBXO38 Regulates Nox1 Stability to Reduce Vascular Endothelial Damage Induced by Low Oscillatory Shear Stress

**DOI:** 10.1155/cdr/4506032

**Published:** 2025-04-23

**Authors:** Wan-li Yu, Li-wen Deng, Huan-huan Li, Chun-kai Wang, Xiang-yi Zuo, Zi-chang Wang, Li Meng, Lan-xin Wen, Wan-zhi Zeng, Yu Zhao, Xue-hu Wang

**Affiliations:** Department of Vascular Surgery, The First Affiliated Hospital of Chongqing Medical University, Chongqing, China

**Keywords:** apoptosis, atherosclerosis, FBXO38, loss, Nox1

## Abstract

Oxidative stress and endothelial dysfunction are critical drivers of atherosclerosis, but the mechanisms regulating oxidative stress under disturbed flow conditions remain incompletely understood. The ubiquitin–proteasome system, particularly E3 ubiquitin ligases, may play a pivotal role in modulating these processes. FBXO38, an E3 ligase involved in proteasomal degradation, has been implicated in various physiological pathways, but its role in regulating oxidative stress in endothelial cells is unknown. We hypothesized that FBXO38 mitigates endothelial damage induced by low oscillatory shear stress (LOSS) by promoting the ubiquitin–proteasome–dependent degradation of Nox1, a major source of reactive oxygen species (ROS). Using an in vitro LOSS model in human umbilical vein endothelial cells (HUVECs) and an in vivo mouse partial carotid ligation model, we assessed the expression of FBXO38 and Nox1 through quantitative PCR, western blotting, immunofluorescence, and immunohistochemistry. LOSS significantly reduced FBXO38 protein expression (by ~60%, *p* < 0.0001 at 24 h), leading to increased Nox1 protein levels (approximately two-fold, *p* < 0.001) and apoptosis. FBXO38 overexpression markedly attenuated Nox1 accumulation (~50% reduction, *p* < 0.05), reduced ROS production, and improved cell viability under LOSS conditions, whereas FBXO38 knockdown exacerbated these effects. Moreover, FBXO38 directly interacted with Nox1, suggesting a ubiquitin-dependent degradation mechanism. Our results reveal that FBXO38 regulates endothelial oxidative stress by controlling Nox1 stability under disturbed shear stress conditions. Although FBXO38 emerges as a promising candidate for therapeutic targeting, further studies are necessary to validate its potential in preclinical and clinical settings.

## 1. Introduction

Oxidative stress and endothelial dysfunction are critical drivers of atherosclerosis (AS), a chronic inflammatory disease characterized by plaque formation, vascular remodeling, and impaired endothelial function [1, 2]. Disturbed blood flow, especially low oscillatory shear stress (LOSS), exacerbates endothelial dysfunction by promoting excessive production of reactive oxygen species (ROS), inflammation, and apoptosis, significantly accelerating lesion formation [3–5]. In contrast, laminar shear stress exerts protective effects, underscoring the critical influence of flow patterns on endothelial cell function [4]. Regions exposed to low shear stress are particularly susceptible to elevated ROS levels, which enhance lipid peroxidation, inflammatory cell recruitment, and apoptosis, further aggravating AS progression [6, 7]. Among ROS-producing enzymes, NADPH oxidases (Noxs), particularly Nox1 and Nox4, have been identified as pivotal mediators of oxidative stress in endothelial cells under disturbed flow conditions, making them attractive targets for therapeutic intervention [8–10].

Protein homeostasis regulated by the ubiquitin–proteasome system (UPS) is also crucial in maintaining cardiovascular health, influencing processes such as inflammation, proliferation, apoptosis, and oxidative stress responses—all fundamental in the pathogenesis of AS [11–13]. Dysfunction of the UPS exacerbates oxidative damage by impairing the degradation of oxidatively modified proteins, thus perpetuating ROS accumulation and endothelial injury [14–16]. Consequently, modulation of the UPS, especially through targeting specific E3 ubiquitin ligases, is emerging as a potential therapeutic approach for mitigating vascular diseases [17, 18].

FBXO38, a member of the F-box protein family, interacts with SKP1 and Cullin-1 to form SCF-type E3 ubiquitin ligase complexes, mediating selective ubiquitination and subsequent proteasomal degradation of target proteins [19, 20]. Previous studies have shown FBXO38's role in regulating the stability of membrane proteins involved in immune checkpoints, such as PD-L1 and PD-1, indicating its potential in modulating inflammation and immune responses [20, 21]. However, the physiological and pathological functions of FBXO38 in the cardiovascular system remain largely unexplored, especially regarding its involvement in oxidative stress regulation and endothelial function. Given the emerging evidence linking other F-box proteins (such as FBXW7) to vascular oxidative stress regulation, we hypothesized that FBXO38 may regulate endothelial oxidative stress and injury under LOSS by targeting Nox1 for ubiquitin–proteasome–dependent degradation [22].

Although the importance of Nox1-derived ROS in endothelial dysfunction is well established, the precise mechanisms controlling Nox1 protein stability under disturbed shear stress remain unclear. Addressing this knowledge gap, we investigated whether and how FBXO38 regulates Nox1 stability and oxidative stress in endothelial cells under LOSS conditions, aiming to reveal novel insights into the mechanistic links between mechanical stimuli, oxidative stress, and endothelial injury in AS.

## 2. Methods and Materials

This study was approved by the Ethics Committee of the First Affiliated Hospital of Chongqing Medical University (Approval No. 2021-616). Animal experiments are conducted in strict accordance with the provisions of the Regulations on the Administration of Experimental Animals. To validate the specificity of the FBXO38 and Nox1 antibody, western blot analysis was performed using FBXO38-knockout and blank control HUVECs. Immunofluorescence (IF) staining was conducted to verify subcellular localization, and immunoprecipitation followed by mass spectrometry confirmed target binding specificity. Each experiment was repeated a minimum of three times.

### 2.1. Cell Lines

Human umbilical vein endothelial cells (HUVECs) were obtained from Procell Life Science & Technology Co., Ltd. (Wuhan, China). Cells were maintained in RPMI-1640 complete medium supplemented with 10% fetal bovine serum (FBS) and 1% penicillin–streptomycin (Procell), cultured at 37°C in a 5% CO₂ humidified incubator. Cells were subcultured upon reaching 90%–95% confluence.

### 2.2. LOSS System

A rocking “see-saw” system (SK-R1807-S, DLAB Scientific, Beijing, China) was employed to simulate LOSS conditions in vitro, applying shear stress approximately ± 4 dyn/cm^2^ as previously described [23]. HUVECs were plated in 6-well plates at 2 × 10^4^ cells per well. Once cells reached ~90% confluence, plates were incubated on the shaker (60 cycles/min) for designated time points (6, 12, and 24 h). Static cultured cells served as controls. These shear stress parameters and exposure duration were chosen based on their physiological relevance and established protocols [24, 25].

### 2.3. qRT-PCR

Total RNA extraction and cDNA synthesis were performed using the SteadyPure Universal RNA Extraction Kit and Evo M-MLV RT Mix Kit (Accurate Biotechnology, Hunan, China). qPCR was conducted with the SYBR Green Premix Pro Taq HS qPCR Kit (Accurate Biotechnology). Relative mRNA levels were quantified using the 2^−^*ΔΔ*Ct method as previously described [26]. Primer sequences are listed in Table 1.

After repeated verification, the expression changes of target genes and proteins in HUVECs peaked following 24-h LOSS treatment. Therefore, HUVECs treated with LOSS for 24 h were selected as the experimental group, while those maintained under static conditions were used as the control group for further investigation.

### 2.4. Western Blot

Proteins were extracted using RIPA buffer (Beyotime, China) with protease/phosphatase inhibitors (100:1:2). After SDS-PAGE separation, proteins were transferred onto PVDF membranes, blocked with 5% skim milk (90 min), and incubated overnight at 4°C with primary antibodies (FBXO38 (Bioss, bs-9122R, 1:1000), Nox1 (UpingBio, YP-Ab-02895, 1:1000), Bax (Abmart, T40051, 1:1000), Bcl-2 (Wanleibio, WL01556, 1:1000), cleaved-caspase3 (Affinitybio, AF7022, 1:1000), and *β*-actin (HUABio, ET1702-52, 1:10000)). Protein bands were visualized by ECL reagent and quantified with ImageJ software according to standard protocols [27, 28].

### 2.5. Coimmunoprecipitation (Co-IP)

Cells were lysed in IP buffer containing protease/phosphatase inhibitors. Lysates were immunoprecipitated overnight (4°C) with agarose-conjugated FBXO38 antibody (Bioss, bs-9122R, 1:1000) or IgG control. After washing, samples were eluted in loading buffer and analyzed by Western blot. Co-IP procedures followed established protocols [29].

### 2.6. ROS Assay

ROS levels were detected using the DCFH-DA fluorescent probe (Beyotime, China), incubated (20 min) at 0.01 mM. Fluorescent images were captured using a Nikon fluorescence microscope. ROS detection methods followed standard procedures [30].

### 2.7. Animal Experiment and Partial Carotid Ligation

This study was approved by the Ethics Committee of the First Affiliated Hospital of Chongqing Medical University (Approval No. 2021-616). A total of 5 male C57BL/6J mice (*n* = 5), aged 6–8 weeks and weighing 22 ± 2 g, were randomly selected and purchased from the Animal Experiment Center of Chongqing Medical University. The mice were housed in SPF conditions at 24°C with 40%–70% humidity. They were maintained on a 12-h light/dark cycle with free access to food and water. The experiment was initiated after 1 week of acclimatization. The self-control design was adopted in the experiment, and each mouse underwent surgical procedures related to the bilateral common carotid arteries. For the surgical procedure, the mice were anesthetized with an intraperitoneal injection of sodium pentobarbital (60 mg/kg), followed by disinfection. A ventral midline incision (4–5 mm) was made in the neck. After the left common carotid artery (LCCA) was exposed through blunt dissection, three of the four caudal branches of the LCCA (the left external carotid artery, the internal carotid artery, and the occipital artery) were ligated with 6-0 silk sutures, while the superior thyroid artery remained intact, forming the surgical group. The right common carotid artery (RCCA) was only bluntly dissected as the sham operation group (*n* = 5), as previously described [31]. After surgery, the incisions were sutured using Tissue-Mend (Veterinary Products Laboratories). Within 24 h after the surgery, the animals were monitored postoperatively for signs of pain or distress, provided analgesics as necessary, and maintained for 28 weeks. The 28-week time point was selected based on previous studies showing significant pathological changes at this duration [32].

At the end of the experiment, the mice were euthanized by cervical dislocation under anesthesia with sodium pentobarbital (100–150 mg/kg). The LCCA and RCCA were harvested, and surrounding fat tissue was carefully removed. The surgery and data collection were carried out strictly in accordance with the principle of researcher blinding.

### 2.8. Hematoxylin-Eosin (HE) and Immunohistochemistry (IHC)

HE staining of vascular tissue was performed according to the classical method [33]. Vascular tissue samples were fixed with 4% paraformaldehyde, embedded with paraffin wax, and then cut into 4 *μ*m slices. After blocking, the sections were stained with primary antibodies targeting FBXO38 (Bioss, bs-9122R, 1: 400) and Nox1 (UpingBio, YP-Ab-02895, 1:400), followed by microscopic imaging. ImageJ software was used for image processing, and lumen area and mean optical density were analyzed for statistical purposes [34, 35].

### 2.9. IF

Cells were fixed (4% PFA), blocked (BSA), incubated overnight (4°C) with FBXO38 (Bioss, bs-9122R) and Nox1 (Abcam, ab121009) antibodies, and stained with fluorescent secondary antibodies and DAPI. Images were analyzed by ImageJ as previously described [36].

### 2.10. Confocal Microscopy

Tissue sections were labeled with 4⁣′,6-diamidino-2-phenylindole (DAPI) and *α*-smooth muscle actin (*α*-SMA). Digital images were obtained via a confocal laser scanning microscope (LSM 710, Carl Zeiss).

### 2.11. Flow Cytometry

Apoptosis was assessed using the Annexin V-FITC/PI apoptosis kit (Beyotime) and analyzed by flow cytometry as per established methods [37].

### 2.12. Small Interfering RNA (siRNA) and Plasmid Transfection

Cells were transfected with FBXO38 siRNA or overexpressed plasmids using Lipofectamine 8000 (Beyotime). HUVECs in 6-well plates (for Western blot or qRT-PCR) were incubated with serum-free OPTI-MEM medium and transfected with FBXO38 siRNA or plasmids at a final concentration of 100 nM. After 24 h of transfection, the cells were collected for further experiments. siRNA sequences and plasmid primers are provided in Tables 2 and 3. Transfection efficiency and time (24 h) were chosen according to standard guidelines and preliminary optimization experiments [38].

### 2.13. Small Guide RNA (sgRNA)

As previously described, Nox1 sgRNA (GenePharma) was transfected into the cells using Lipofectamine 8000 (Beyotime), and the verification was carried out 24 h after the transfection [38]. The sgRNA sequences are illustrated in Table 4.

### 2.14. Statistical Analysis

Statistical analyses used GraphPad Prism 8.0 (California, United States). Data (mean ± *S*EM, *n* ≥ 3 independent experiments) were analyzed by Student's *t*-test (two-group comparison) or one-way ANOVA (multigroup). Statistical significance: ⁣^∗^*p* < 0.05, ⁣^∗∗^*p* < 0.01, ⁣^∗∗∗^*p* < 0.001, ⁣^∗∗∗∗^*p* < 0.0001.

## 3. Results

### 3.1. FBXO38 Expression Is Significantly Reduced in HUVECs Under LOSS Conditions

**Figure 1:**
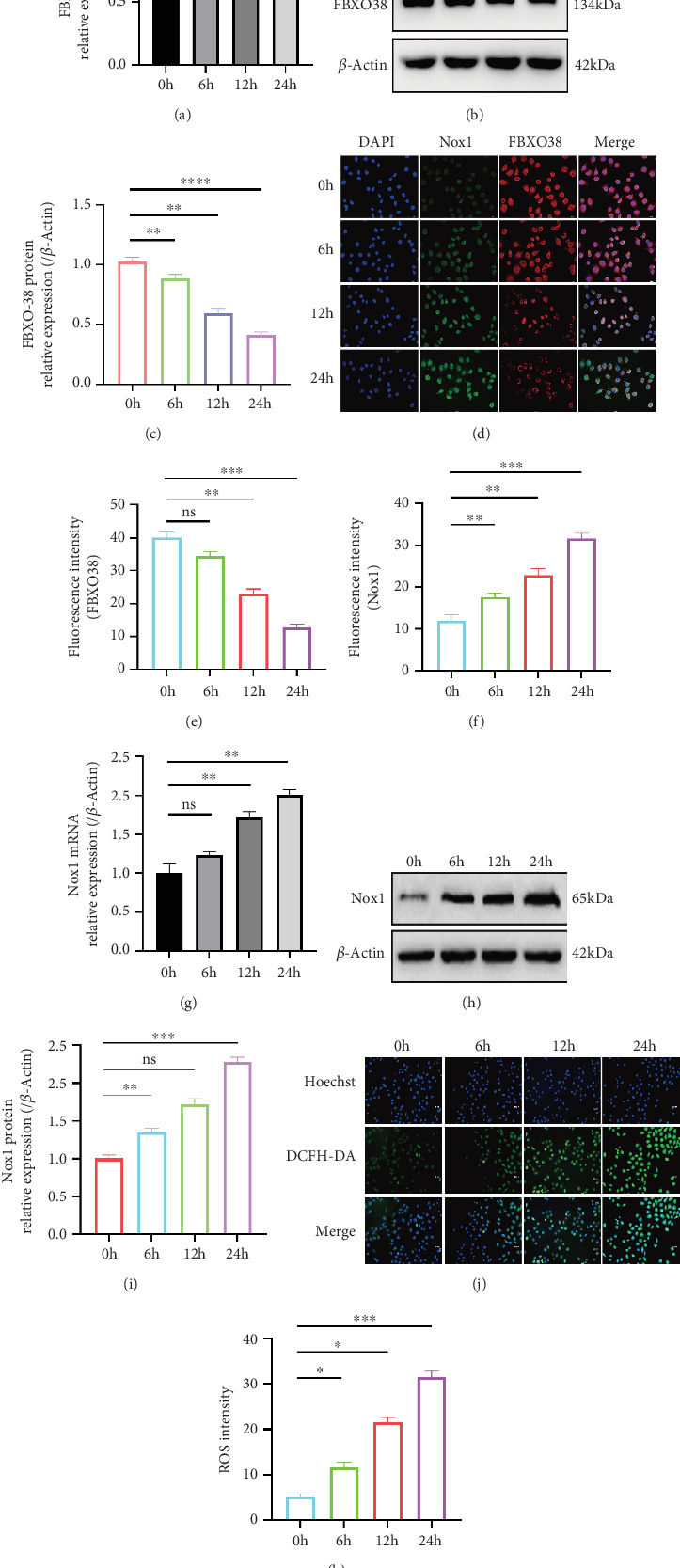
Under low oscillatory shear stress (LOSS), FBXO38 expression decreases while Nox1 expression and ROS levels increase over time in HUVECs. (a) qPCR analysis of FBXO38 mRNA in HUVECs exposed to LOSS for 0, 6, 12, or 24 h (⁣^∗^*p* < 0.05; ⁣^∗∗^*p* < 0.01; *n* = 3). (b, c) Western blot detection and quantification of FBXO38 protein levels under the same conditions (⁣^∗∗^*p* < 0.01; ⁣^∗∗∗∗^*p* < 0.0001; *n* = 3). (d) Representative immunofluorescence images showing FBXO38 (red) and Nox1 (green) at 0, 6, 12, and 24 h after LOSS (magnification: ×20, scale bar = 1000 *μ*m). (e, f) Fluorescence intensity quantification of FBXO38 and Nox1, respectively, (ns, *p* > 0.05; ⁣^∗∗^*p* < 0.01; ⁣^∗∗∗^*p* < 0.001; *n* = 3). (g) qPCR analysis of Nox1 mRNA in HUVECs following LOSS (ns, *p* > 0.05; ⁣^∗∗^*p* < 0.01; *n* = 3). (h, i) Western blot detection and quantification of Nox1 protein (ns, *p* > 0.05; ⁣^∗∗^*p* < 0.01; ⁣^∗∗∗^*p* < 0.001; *n* = 3). (j) Representative images of ROS staining (HUVECs treated for 0, 6, 12, or 24 h under LOSS; magnification: ×20, scale bar = 1000 *μ*m). (k) Quantification of ROS fluorescence intensity (⁣^∗^*p* < 0.05; ⁣^∗∗∗^*p* < 0.001; *n* = 3). Data are presented as mean ± SEM from three independent experiments (*n* = 3). Significance is determined by one-way ANOVA with Tukey's post hoc test (unless otherwise specified). “ns” indicates no statistical significance.

Using a rocker system to simulate LOSS conditions in HUVECs, we observed that FBXO38 mRNA and protein expression levels were significantly reduced in a time-dependent manner [23, 24]. At 24 h, FBXO38 mRNA decreased by approximately 40% (*p* < 0.05) and protein levels by around 60% (*p* < 0.0001) compared with static controls (Figures 1a, 1b, and 1c). IF analysis confirmed the reduction, revealing decreased FBXO38 staining intensity at 12 h and 24 h of LOSS treatment (Figure 1d,e).

### 3.2. LOSS Induces Nox1 Upregulation and Nox-Dependent Oxidative Stress

Consistent with previous studies indicating LOSS-induced oxidative stress via Nox enzymes [39], our results showed that after the action of LOSS for 24 h, the levels of Nox1 mRNA (~2-fold, *p* < 0.01) and protein (~2.5-fold, *p* < 0.001) in HUVECs increased to the peak values (Figures 1g, 1h, and 1i). Nox1 and ROS levels, measured by IF and DCFH-DA fluorescence, significantly increased (about 5.5-fold, *p* < 0.001) at 24 h compared with static controls (Figures 1d, 1f, 1j, and 1k).

### 3.3. Apoptosis in HUVECs Gradually Increases Under LOSS

**Figure 2:**
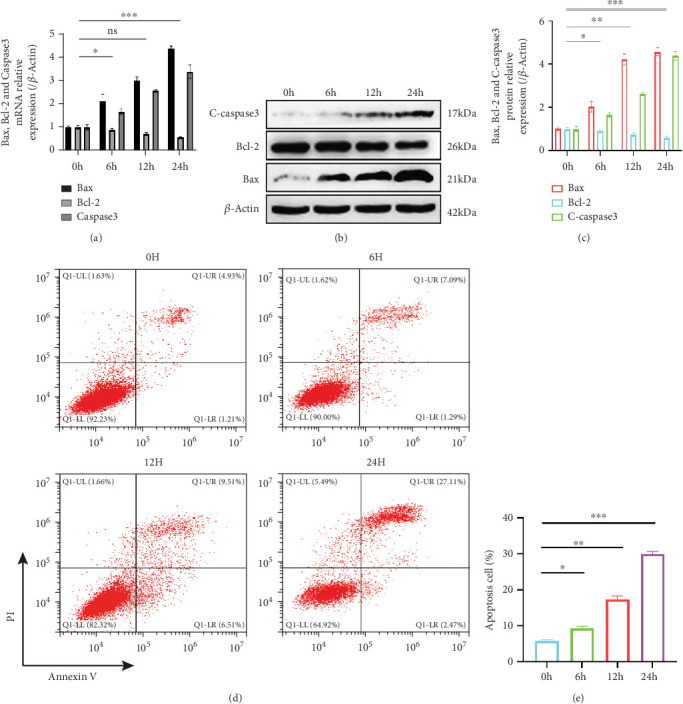
Under LOSS, the level of apoptosis increases over time in HUVECs. (a) qPCR analysis of Bax, Bcl-2, and Caspase3 mRNA in HUVECs exposed to LOSS for 0, 6, 12, or 24 h (ns, *p* > 0.05; ⁣^∗^*p* < 0.05; ⁣^∗∗∗^*p* < 0.001; *n* = 3). (b, c) Western blot detection and quantification of Bax, Bcl-2, and C-caspase3 protein levels under the same conditions (⁣^∗^*p* < 0.05; ⁣^∗∗^*p* < 0.01; ⁣^∗∗∗^*p* < 0.001; *n* = 3). (d, e) Flow cytometry analysis of the apoptosis of HUVECs under the same conditions; the data are expressed as mean ± SEM (⁣^∗^*p* < 0.05; ⁣^∗∗^*p* < 0.01; ⁣^∗∗∗^*p* < 0.001; *n* = 3).

Given the crucial role of apoptosis in AS [40, 41], we evaluated apoptotic markers under LOSS. Bax protein expression increased significantly (~4.5-fold at 24 h, *p* < 0.001), cleaved-caspase3 was elevated approximately 4.2-fold (*p* < 0.001), and antiapoptotic protein Bcl-2 expression decreased by about 50% (*p* < 0.001) at 24 h (Figures 2a, 2b, and 2c). Flow cytometry analysis demonstrated an increase in apoptotic cell proportion from baseline (6.6%) to approximately 32.6% (*p* < 0.001) after 24 h of LOSS (Figure 2d,e).

### 3.4. Overexpression of FBXO38 Significantly Attenuates LOSS-Induced Oxidative Stress and Apoptosis

**Figure 3:**
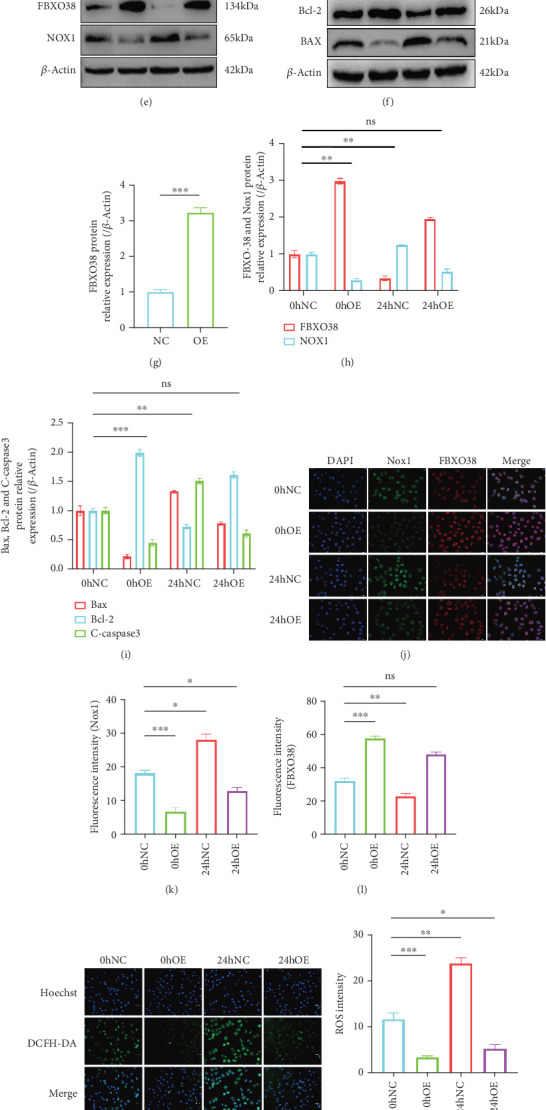
FBXO38 overexpression attenuates LOSS-induced oxidative stress and apoptosis. HUVECs with over-expressed FBXO38 (OE group) and negative control group (NC group) are established, respectively, for subsequent experiments. (a) qPCR analysis of FBXO38 mRNA in HUVECs of NC group and OE group (⁣^∗∗^*p* < 0.01; *n* = 3). (d, g) Western blot detection and quantification of FBXO38 protein levels in the abovementioned two groups of HUVECs (⁣^∗∗∗^*p* < 0.001; *n* = 3). (b, c) qPCR analysis of FBXO38, Nox1, Bax, Bcl-2, and Caspase3 mRNA in two groups of HUVECs exposed to LOSS for 0 and 24 h (ns, *p* > 0.05; ⁣^∗^*p* < 0.05; ⁣^∗∗^*p* < 0.01; *n* = 3). (e, f, h, i) Western blot detection and quantification of FBXO38, Nox1, Bax, Bcl-2, and C-caspase3 protein levels under the same conditions (ns, *p* > 0.05; ⁣^∗∗^*p* < 0.01; ⁣^∗∗∗^*p* < 0.001; *n* = 3). (j) Representative immunofluorescence images showing FBXO38 (red) and Nox1 (green) in two groups of HUVECs at 0 and 24 h after LOSS (magnification: ×20, scale bar = 1000 *μ*m). (k, l) Fluorescence intensity quantification of FBXO38 and Nox1, respectively (ns, *p* > 0.05; ⁣^∗^*p* < 0.05; ⁣^∗∗^*p* < 0.01; ⁣^∗∗∗^*p* < 0.001; *n* = 3). (m) Representative images of ROS staining (two groups of HUVECs treated for 0 and 24 h under LOSS; magnification: ×20, scale bar = 1000 *μ*m). (n) Quantification of ROS fluorescence intensity (⁣^∗^*p* < 0.05; ⁣^∗∗^*p* < 0.01; ⁣^∗∗∗^*p* < 0.001; *n* = 3). (o, p) Flow cytometry analysis the apoptosis of HUVECs under the same conditions (ns, *p* > 0.05; ⁣^∗^*p* < 0.05; ⁣^∗∗∗^*p* < 0.001; *n* = 3). Data are presented as mean ± SEM from three independent experiments (*n* = 3). Significance is determined by one-way ANOVA with Tukey's post-hoc test (unless otherwise specified). “ns” indicates no statistical significance.

Given the downregulation of FBXO38 and concurrent increases in Nox1, ROS, and apoptosis under LOSS, we hypothesized FBXO38 negatively regulates these events. FBXO38 overexpression (verified by ~3-fold mRNA increase, *p* < 0.01; protein levels increased approximately 3.3-fold, *p* < 0.001) significantly reduced LOSS-induced elevations in Nox1 (about 50% reduction, *p* < 0.01), Bax (~60% reduction, *p* < 0.001), and cleaved-caspase3 (~45% reduction, *p* < 0.001), and restored Bcl-2 (~70% increase, *p* < 0.001) (Figures 3a, 3b, 3c, 3d, 3e, 3f, 3g, 3h, and 3i). Furthermore, IF, ROS assay, and flow cytometry confirm that FBXO38 overexpression lowers intracellular ROS and apoptosis (~60% reduction, *p* < 0.001), indicating a protective effect under LOSS-induced oxidative stress (Figures 3j, 3k, 3l, 3m, 3n, 3o, and 3p).

### 3.5. FBXO38 Knockdown Exacerbates LOSS-Induced Oxidative Stress and Apoptosis

**Figure 4:**
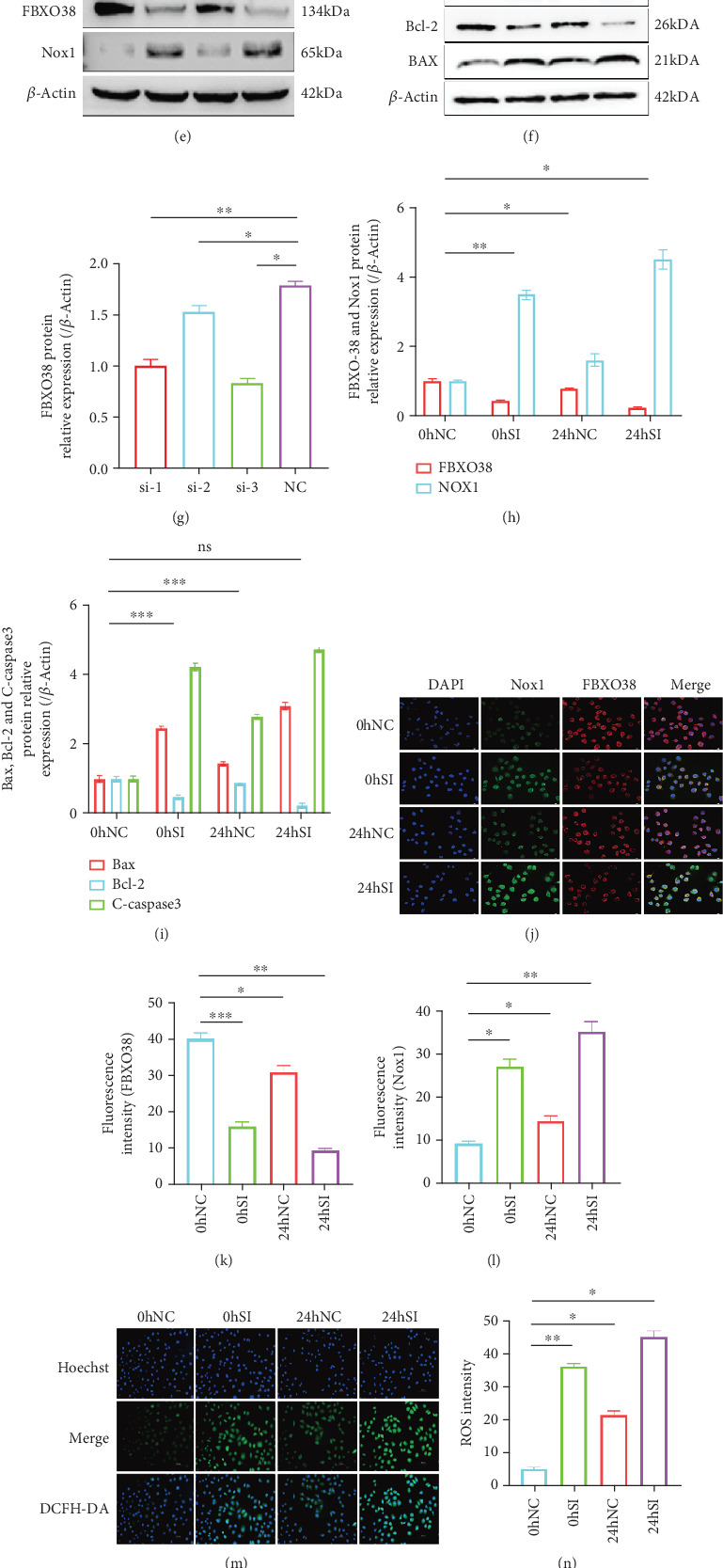
FBXO38 knockdown exacerbates LOSS-induced oxidative stress and apoptosis. (a) qPCR analysis of FBXO38 mRNA in HUVECs after transfecting different siRNA of FBXO38 (⁣^∗^*p* < 0.05; ⁣^∗∗^*p* < 0.01; *n* = 3). (d, g) Western blot detection and quantification of FBXO38 protein levels in HUVECs after transfecting different siRNA of FBXO38 (⁣^∗^*p* < 0.05; ⁣^∗∗^*p* < 0.01; *n* =  3). After verification, HUVECs transfected with SiRNA-3 are selected as the knockdown group (Si) and used with the negative control group (NC) for subsequent experiments. (b, c) qPCR analysis of FBXO38, Nox1, Bax, Bcl-2, and Caspase3 mRNA in two groups of HUVECs exposed to LOSS for 0 and 24 h (⁣^∗^p < 0.05; ⁣^∗∗^p < 0.01; ⁣^∗∗∗^*p* < 0.001; *n* = 3). (e, f, h, i) Western blot detection and quantification of FBXO38, Nox1, Bax, Bcl-2, and C-caspase3 protein levels under the same conditions (ns, *p* > 0.05; ⁣^∗^*p* < 0.05; ⁣^∗∗^*p* < 0.01; ⁣^∗∗∗^*p* < 0.001; *n* = 3). (j) Representative immunofluorescence images showing FBXO38 (red) and Nox1 (green) in two groups of HUVECs at 0 and 24 h after LOSS (magnification: ×20, scale bar = 1000 *μ*m). (k, l) Fluorescence intensity quantification of FBXO38 and Nox1, respectively (⁣^∗^*p* < 0.05; ⁣^∗∗^*p* < 0.01; ⁣^∗∗∗^*p* < 0.001; *n* = 3). (m) Representative images of ROS staining (two groups of HUVECs treated for 0 and 24 h under LOSS; magnification: ×20, scale bar = 1000 *μ*m). (n) Quantification of ROS fluorescence intensity (⁣^∗^*p* < 0.05; ⁣^∗∗^*p* < 0.01; *n* = 3). (o, p) Flow cytometry analysis the apoptosis of HUVECs under the same conditions (⁣^∗^*p* < 0.05; ⁣^∗∗^*p* < 0.01; *n* = 3). Data are presented as mean ± SEM from three independent experiments (*n* = 3). Significance is determined by one-way ANOVA with Tukey's post hoc test (unless otherwise specified). “ns” indicates no statistical significance.

In contrast, siRNA-mediated knockdown of FBXO38 (verified by ~65% reduction in protein level, *p* < 0.05) further amplified Nox1 expression (~2.5-fold higher than LOSS alone, *p* < 0.01), increased Bax (~2.2-fold, *p* < 0.001), cleaved-caspase3 (~4-fold, *p* < 0.001), and reduced Bcl-2 (~50% decrease, *p* < 0.001). Flow cytometry showed increased apoptosis (from ~15% in LOSS alone to ~46% with FBXO38 knockdown, *p* < 0.01), and ROS production also rose significantly (~1.6-fold increase, *p* < 0.05) (Figures 4a, 3b, 3c, 3d, 3e, 3f, 3g, 3h, 3i, 3j, 3k, 3l, 3m, 3n, 3o, and 4p).

### 3.6. Nox1 Knockout Partially Reverses Oxidative Stress and Apoptosis Induced by FBXO38 Deficiency

**Figure 5:**
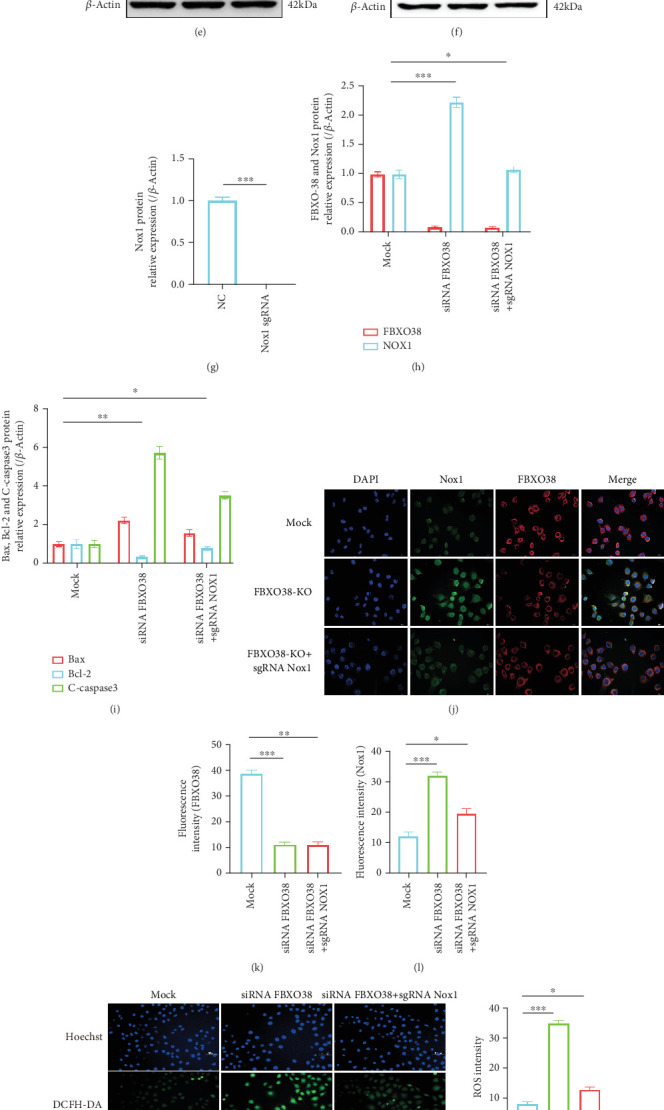
Knockdown of Nox1 reverses elevated ROS and apoptosis in FBXO38-deficient HUVECs. (a) qPCR analysis of Nox1 mRNA in HUVECs after transfecting sgRNA of Nox1 (⁣^∗∗∗^*p* < 0.001; *n* = 3). (d, g) Western blot detection and quantification of Nox1 protein levels in HUVECs after transfecting sgRNA of Nox1 (⁣^∗∗∗^*p* < 0.001; *n* = 3). Set up the siRNA FBXO38 group, the siRNA FBXO38 + sgRNA Nox1 group, and the blank control group (Mock group) for subsequent experiments. (b, c) qPCR analysis of FBXO38, Nox1, Bax, Bcl-2, and Caspase3 mRNA in HUVECs of the above three groups.(⁣^∗^*p* < 0.05; ⁣^∗∗^*p* < 0.01; *n* = 3). (e, f, h, i) Western blot detection and quantification of FBXO38, Nox1, Bax, Bcl-2, and C-caspase3 protein levels under the same conditions (⁣^∗^*p* < 0.05; ⁣^∗∗^*p* < 0.01; ⁣^∗∗∗^*p* < 0.001; *n* = 3). (j) Representative immunofluorescence images showing FBXO38 (red) and Nox1 (green) under the same conditions (magnification: ×20, scale bar = 1000 *μ*m). (k, l) Fluorescence intensity quantification of FBXO38 and Nox1, respectively (⁣^∗^*p* < 0.05; ⁣^∗∗^*p* < 0.01; ⁣^∗∗∗^*p* < 0.001; *n* = 3). (m) Representative images of ROS staining (three groups of HUVECs under the same conditions as above; magnification: ×20, scale bar = 1000 *μ*m). (n) Quantification of ROS fluorescence intensity (⁣^∗^*p* < 0.05; ⁣^∗∗∗^*p* < 0.001; *n* = 3). (o, p) Flow cytometry analysis the apoptosis of HUVECs under the same conditions as above (⁣^∗^*p* < 0.05; ⁣^∗∗^*p* < 0.01; *n* = 3). Data are presented as mean ± SEM from three independent experiments (*n* = 3). Significance is determined by one-way ANOVA with Tukey's post hoc test (unless otherwise specified).

To verify whether Nox1 mediates increased oxidative stress and apoptosis observed with FBXO38 deficiency, we employed sgRNA-mediated Nox1 knockout, achieving a ~95% decrease in Nox1 levels (*p* < 0.001) (Figures 5a, 5d, and 5g). Compared with FBXO38-knockdown cells, Nox1 knockout significantly reduced Bax (~40% reduction, *p* < 0.05), cleaved-caspase3 (~50% reduction, *p* < 0.05), ROS (~65% reduction, *p* < 0.001), and restored Bcl-2 (~60% increase, *p* < 0.05) (Figures 5b, 5c, 5e, 5f, 5h, 3i, 3j, 3k, 3l, 3m, 3n, 3o, and 4p).

### 3.7. FBXO38 Directly Interacts and Colocalizes With Nox1

**Figure 6:**
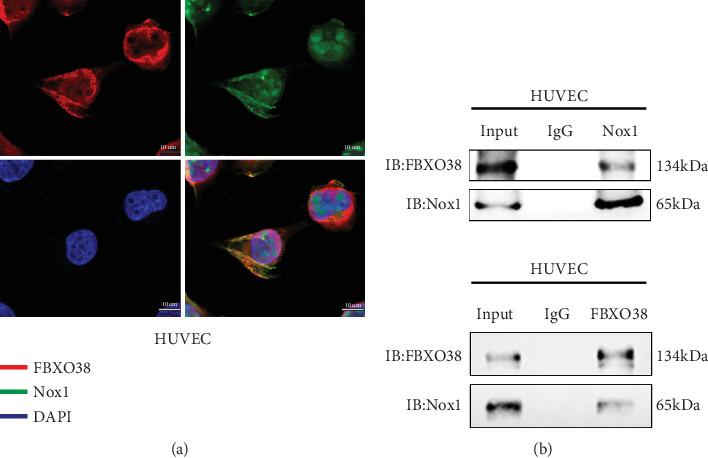
FBXO38 and Nox1 Interact and Co-localize. (a) Use immunofluorescence staining to analyze the co-localization of FBXO38 and Nox1 proteins in the cytoplasm of HUVECs. Stain the cell nuclei with DAPI. Magnification: ×240, scale bar = 10 *μ*m. (b) Use immunocoprecipitation to evaluate the interaction between FBXO38 and Nox1.

IF analysis revealed significant cytoplasmic colocalization of FBXO38 and Nox1 in HUVECs. Co-IP assays further confirmed direct physical interaction between FBXO38 and Nox1 proteins, supporting the hypothesis of direct ubiquitination-mediated regulation (Figure 6a,b). However, direct ubiquitination assays were not performed and will be addressed in future experiments.

### 3.8. In Vivo Validation: Reduced FBXO38 Expression Is Associated With Increased Nox1 and Intimal Hyperplasia

**Figure 7:**
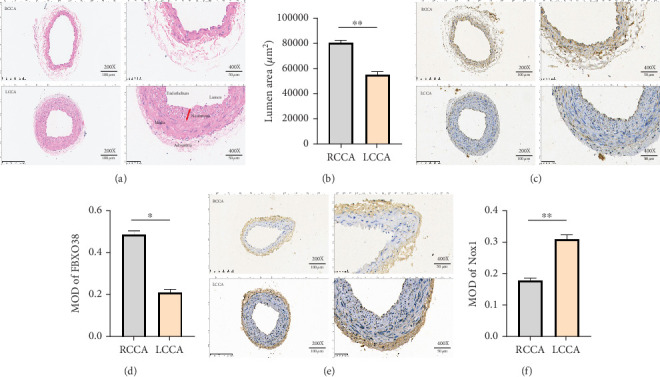
In vivo, FBXO38 expression is negatively correlated with LOSS-mediated oxidative stress and intimal hyperplasia. (a, b) Left and right common carotid artery sections and HE staining (magnification: ×200; ×400, scale bar = 100 *μ*m; 50 *μ*m), and lumen area is quantitatively analyzed (⁣^∗∗^*p* < 0.01; *n* = 5). (c, d) LCCA and RCCA immunohistochemical staining (magnification: ×200; ×400, scale bar = 100 *μ*m; 50 *μ*m), and FBXO38 in the endothelium quantitative analysis (⁣^∗^*p* < 0.05, *n* = 5). (e, f) LCCA and RCCA immunohistochemical staining (magnification: ×200; ×400, scale bar = 100 *μ*m; 50 *μ*m), and Nox1 in the endothelium quantitative analysis (⁣^∗∗^*p* < 0.01, *n* = 5). Data are presented as mean ± SEM from five independent experiments (*n* = 5). Significance is determined by one-way ANOVA with Tukey's post hoc test (unless otherwise specified). MOD: average optical density.

In the partial carotid ligation mouse model, the LCCA exhibited significant intimal hyperplasia and reduced lumen area (~35% reduction, *p* < 0.01) at 28 weeks postsurgery compared to sham-operated RCCA controls (Figure 7a,b). IHC showed significantly reduced FBXO38 (~60% decrease, *p* < 0.05) and increased Nox1 (~1.7-fold increase, *p* < 0.01) expression in endothelial regions of LCCA compared to RCCA controls, closely mirroring the in vitro findings (Figures 7c, 7d, 7e, and 7f).

## 4. Discussion

Our study demonstrates for the first time that FBXO38 acts as a critical regulator of endothelial oxidative stress by controlling Nox1 protein stability under conditions of LOSS. We observed that LOSS significantly downregulated FBXO38 expression, leading to increased Nox1 protein accumulation, elevated ROS generation, and enhanced endothelial apoptosis. These findings provide new mechanistic insights into how mechanical stimuli influence the UPS to modulate endothelial function and highlight FBXO38 as a key node in mechanotransduction pathways linked to AS.

Oxidative stress and endothelial dysfunction are widely recognized as fundamental contributors to the initiation and progression of AS [42]. Nox1, a prominent source of ROS in endothelial cells, is strongly implicated in disturbed flow-induced oxidative stress and vascular injury [43, 44]. However, regulatory mechanisms controlling Nox1 protein degradation, particularly under LOSS conditions, remain poorly defined. Previous studies have identified several F-box proteins, such as FBXW7, as regulators of endothelial oxidative stress by promoting ubiquitin-dependent degradation of key signaling proteins [45, 46]. In this context, our identification of FBXO38 as an E3 ligase responsible for controlling Nox1 stability provides novel insights into the molecular regulation of oxidative stress in response to mechanical stress. Although we confirmed direct interactions between FBXO38 and Nox1 through Co-IP and IF, further investigations—such as direct ubiquitination assays—are essential to conclusively demonstrate FBXO38-dependent ubiquitination of Nox1.

Therapeutically, targeting FBXO38-mediated Nox1 degradation holds intriguing potential for mitigating endothelial damage and AS progression. Overexpression of FBXO38 markedly attenuated LOSS-induced oxidative stress and apoptosis, underscoring its protective role. Nevertheless, caution is necessary regarding its clinical translation, since pharmacological or genetic modulation of FBXO38 was not tested in vivo in this study. Therefore, subsequent preclinical studies utilizing FBXO38 knockout or transgenic animal models are essential to thoroughly validate therapeutic efficacy, assess potential off-target effects, and evaluate safety profiles. Exploring small-molecule modulators, gene-editing strategies, or proteasome-targeted therapies directed at FBXO38 may further elucidate its translational potential and limitations.

Mechanistically, while our findings indicate that FBXO38 physically associates with Nox1, conclusive demonstration of direct ubiquitination remains lacking. Future studies involving in vitro ubiquitination assays, mass spectrometry identification of ubiquitination sites, and proteasome inhibition experiments are critical for definitively establishing whether FBXO38 directly catalyzes ubiquitination and subsequent proteasomal degradation of Nox1. Additionally, we cannot exclude the possibility that FBXO38 cooperates with other E3 ligases or adaptor proteins to modulate Nox1 stability, which warrants further investigation.

Another important consideration involves alternative compensatory mechanisms in endothelial cells, such as NF-*κ*B and Nrf2 signaling pathways, which may compensate for reduced FBXO38 expression under LOSS conditions. Exploring these alternative pathways will offer a more comprehensive understanding of how endothelial cells maintain redox homeostasis when FBXO38 activity is impaired.

Several limitations of our study merit attention. First, although the LOSS model used in vitro provides useful insights into endothelial responses to disturbed flow, it does not fully recapitulate complex physiological shear stress conditions in vivo. Furthermore, static controls do not entirely reflect physiological laminar shear stress conditions present in healthy vessels. Secondly, animal model validation was limited to partial carotid ligation and did not extend to other clinically relevant vascular beds such as coronary or peripheral arteries. Third, human vascular tissue samples were not investigated, limiting direct translational relevance. Fourth, potential off-target effects of siRNA-mediated knockdown of FBXO38 necessitate verification through alternative genetic methods such as CRISPR/Cas9-based gene editing. Lastly, this study did not address the long-term safety and feasibility of pharmacologically or genetically targeting FBXO38 for therapeutic purposes.

Future research should comprehensively investigate FBXO38's role in regulating vascular oxidative stress and endothelial function across different vascular tissues. Investigations should include detailed in vivo experiments using FBXO38 knockout or transgenic mouse models, evaluation of FBXO38 modulators in preclinical studies, and validation of findings in human clinical samples. Furthermore, identification of specific small-molecule inhibitors or activators, gene-editing strategies, or proteasome-targeting approaches might further elucidate the therapeutic potential and safety profile of modulating FBXO38 activity in clinical scenarios.

In summary, this study identifies FBXO38 as a novel regulator linking disturbed shear stress conditions to endothelial oxidative injury via modulation of Nox1 stability. While our findings offer mechanistic and therapeutic promise, further rigorous validation in animal models and clinical research remains essential before clinical application can be considered. Nevertheless, our results provide an important foundation for future investigations targeting the FBXO38–Nox1 pathway in shear stress-related vascular diseases.

## 5. Conclusion

In summary, our study underscores the pivotal role of FBXO38 in regulating Nox1 stability and mitigating oxidative stress and endothelial apoptosis under LOSS, thereby offering new insights into AS pathogenesis. Given the link between UPS dysfunction and vascular diseases, FBXO38 emerges as a promising therapeutic target for countering disturbed flow-induced vascular damage. Building on these findings, future research should

1. Explore FBXO38 in other vascular diseases: Investigate whether FBXO38's protective mechanisms extend to conditions such as coronary artery disease, peripheral artery disease, or stroke.
2. Develop targeted therapeutics: Pursue small-molecule modulators or gene-editing strategies aimed at restoring or enhancing FBXO38 function, and assess their efficacy in preventing LOSS-induced endothelial injury.
3. Validate clinical relevance: Conduct preclinical and clinical studies to confirm the therapeutic impact of modulating the FBX038-Nox1 axis and to evaluate long-term safety and efficacy in AS management.

By clarifying FBXO38's regulatory network and its broader roles in vascular biology, future investigations may pave the way for novel interventions that reduce oxidative stress, preserve endothelial function, and slow the progression of AS.

## Figures and Tables

**Table 1:** Primer sequences used for qRT-PCR.

**Gene**	**Forward primer(5**⁣′**-3**⁣′**)**	**Reverse primer(5**⁣′**-3**⁣′**)**
FBXO38	GGTGGCCGAGAGTGGAAATAATA	TTACTACACGCTGAAGACCACTG
Nox1	CCTCCATTCTCTCCAGCCTATCT	AACTCCTCCGGATGAACTCAGTA
Bax	TTGCTTCAGGGTTTCATCCA	AGACACTCGCTCAGCTTCTTG
Bcl-2	TGGCCAGGGTCAGAGTTAAA	TGGCCTCTCTTGCGGAGTA
Caspase3	CATGGAAGCGAATCAATGGACT	CTGTACCAGACCGAGATGTCA
*β*-actin	CATGTACGTTGCTATCCAGGC	CTCCTTAATGTCACGCACGAT

**Table 2:** The sense and antisense of siRNA.

**Gene**	**Sense(5**⁣′**-3**⁣′**)**	**Antisense(5**⁣′**-3**⁣′**)**
H81545-siFBXO38-1	GGAUCAGAUGUUUCGUGAATT	UUCACGAAACAUCUGAUCCTT
H81545-siFBXO38-2	GCAGAUAAAUCCACUAGUATT	UACUAGUGGAUUUAUCUGCTT
H81545-siFBXO38-3	CAUCAGCUCUUGUUAGCAATT	UUGCUAACAAGAGCUGAUGTT

**Table 3:** Primer sequences used for plasmid.

**Gene**	**Forward primer(5**⁣′**-3**⁣′**)**	**Reverse primer(5**⁣′**-3**⁣′**)**
PCDH-FBXO38	GCCACCATGGGGCCACGAAAGAAAA	TTAAATGTAGTCATCTTCAACTGGCTC

**Table 4:** The sense of sg-RNA.

**Gene**	**Sense(5**⁣′**-3**⁣′**)**
h⁣^∗^sg-Nox1-1	CACCGTCTCATGATGAGAAAAAGGG

## Data Availability

The data that support the findings of this study are available from the corresponding author upon a reasonable request.
